# Hematology: the specialty with a record number of new approvals

**DOI:** 10.3389/fmed.2024.1385052

**Published:** 2024-02-29

**Authors:** Eleni Gavriilaki

**Affiliations:** 2nd Propedeutic Department of Internal Medicine, Aristotle University of Thessaloniki, Thessaloniki, Greece

**Keywords:** complement, gene therapy, small molecules, biologics, bispecific antibodies, CAR T cells, classical hematology, hematological oncology

Hematology flows from the Greek haimo-, or “blood,” and the Latin logia, or “the study of.” Since blood has been an easy target to study, several eminent figures, also called “fathers” and “mothers” of hematology, have significantly contributed to the success story of this specialty. Over the last 30 years, hematologists have witnessed miracles in multiple fields, such as transplantation that evolved from fresh blood to peripheral stem cells and now cellular or gene therapies; or chronic myeloid leukemia that has been one of the first curable cancer without chemotherapy (1). Tremendous research and development in this unique clinico-laboratory specialty has led to better understanding of multiple disorders and targeted therapies.

In 2023, the Center for Drug Evaluation and Research (CDER) has approved 55 new drugs, with the Center for Biologic Evaluation and Research (CBER) keeping the pace up too. Both centers belong to Federal Drug Association (FDA). These numbers reflect a growing amount of small molecule and biologic pharmacopeia, as well as cell and cellular products. As a therapeutic area, hematology continues to be a leading star, having received the majority of approvals in both areas. Table 1 summarizes selected approvals concerning hematology.

**Table 1:** Selected FDA approvals concerning hematology.

**Biologic name**	**Indication**	**Action**
Pirtobrutinib	Mantle cell lymphoma	BTK inhibitor
Epcoritamab	DLBCL and high-grade B-cell lymphoma	CD20 × CD3 T-cell engager
Glofitamab	DLBLC or large B-cell lymphoma	CD20 × CD3 T-cell engager
Quizartinib	AML	FLT3 kinase inhibitor
Talquetamab	Multiple myeloma	GPRC5D × CD3 T-cell engager
Elranatamab	Multiple myeloma	BCMA × CD3 T-cell engager
Motixafortide	Hematopoietic stem cell mobilization for autologous transplantation in multiple myeloma	CXCR4 inhibitor
Momelotinib	Myelofibrosis in adults with anemia	JAK1/2, ALK2 inhibitor
Efbemalenograstim alfa	Neutropenia	Recombinant leukocyte growth factor
Iptacopan	Paroxysmal nocturnal hemoglobinuria	Complement factor B inhibitor
Ten fusion protein	Hemophilia A	Recombinant antihemophilic factor
Omidubicel	Neutrophil recovery in patients with hematologic malignancies	Nicotinamide modified allogeneic hematopoietic progenitor cell therapy
Valoctocogene roxaparvovec	Hemophilia A	AAV-based gene therapy with modified factor VIII transgene
Anthrax vaccine adsorbed, adjuvanted	Post-exposure prophylaxis	Protective antigen protein-based vaccine
Prothrombin complex concentrate, human	Urgent warfarin reversal	Prothrombin complex concentrate containing factor II, VII, IX and X, and proteins C and S
ADAMTS13, recombinant	cTTP	Recombinant rADAMTS13
Exagamglogene autotemcel	Sickle cell disease	CRISPR–Cas9-based BCL11a-gene-editing therapy
Lovotibeglogene autotemcel	Sickle cell disease	Lentivirus-based gene therapy with HbAT87Q transgene

FDA, Food and Drug Administration; DLBCL, diffuse large B cell lymphoma; AML, acute myeloid leukemia; BCMA, B-cell maturation antigen; TTP, thrombotic thrombocytopenic purpura; B-cell maturation antigen; JAK, Janus kinase; ALK2, Activin receptor-like kinase-2; AAV, Adeno-associated viruses; ADAMTS13, a disintegrin and metalloproteinase with thrombospondin motifs; CRIPSR, clustered regularly interspaced short palindromic repeats.

The most celebrated ones are the first product of gene editing utilizing CRISPR–Cas9 and a burst of gene therapies. In particular, exagamglogene autotemcel is the first gene editor based on CRISPR–Cas8 to be approved from the FDA, for sickle cell disease (SCD). This *ex vivo* gene therapy product (Exa-cel) is genetically modified at BCL11a transcription factor, re-enabling fetal hemoglobin production. In this context, defects in β-hemoglobin are compensated by the therapeutically upregulated fetal hemoglobin. Although clinical data suggests curative potential, further studies are needed to confirm its durability. Another gene therapy, lovotibeglogene autotemcel has been approved for SCD. A lentiviral vector is used to insert a transgene encoding the non-sickling hemoglobin HbAT87Q. Advances in gene editors and small molecules are expected in the near future, with the aim of wider accessibility (2).

Another celebrated field is the complement system, with full approvals to four inhibitors in 2023, concerning hematology and other specialties (3). Three of them target the terminal complement C5, which has been the target of eculizumab. This first-in-class complement inhibitor has been approved since 2007, for paroxysmal nocturnal hemoglobinuria (PNH), an ultra-rare hematologic disorder. In 2023, an RNA aptamer, avacincaptad pegol, targeting C5 has been approved for an ocular disease. Another monoclonal antibody against C5 is pozelimab. In 2023, pozelimab has been approved for CHAPLE (CD55-deficient protein-losing enteropathy), expands the horizon of complement inhibitors. New horizons in the field open with the first oral monotherapy, iptacopan, a Factor B inhibitor being approved for PNH (4). Additional complement competition is expected soon, including another oral complement inhibitor against factor D (5).

Interestingly, both gene therapy and complement inhibition are fields enabled by academic discoveries, highlighting that academic and clinical researchers have been the critical driving force to navigate therapeutics out of the shadow into the spotlight (6). These advancing fields strengthen the role of “classical hematology” as a continuously evolving field. In this field, great developments have been also seen in thrombotic microangiopathies, including thrombotic thrombocytopenic purpura (TTP) (7). This year marked the first drug approval in congenital TTP.

Beyond them, researchers and physicians working within the field of hematology are also actively involved in developments of relative fields, such as infectious diseases, including COVID-19 and vaccine development (8). More notable new entrants into the pharmacopeia and expansion of indications are also expected in the coming years.

Last but not least, hematological oncology continues to thrive with novel approvals and revolutionary therapies. Momelotinib has received approval from myelofibrosis patients, covering an unmet clinical need in the subset of patients with anemia (9). In parallel, bispecific antibodies and CAR (Chimeric antigen receptors)-T cells expand their products and indications mainly in patients with lymphoproliferative diseases and multiple myeloma (10). Talquetamab and elranatamab are two of these drugs approved in 2023 for multiple myeloma (11).

Nevertheless, novel developments come together with long-lasting challenges in the field. The most important one for our patients being accessibility to innovating treatments. Another one is diversity, equity and inclusion in research and clinical purposes (12). Finally, in the era of precision medicine, personalized selection of patients that would benefit from these treatments is important. Overcoming these challenges necessitates coordinated efforts from every partner in this field, including patient's organizations.

In Conclusion, hematology covers a broad spectrum of exciting advances that pave the way for a brighter future for our young colleagues and most importantly, for our patients. It is our role to continuously work for the success of our field.

## Author contributions

EG: Writing—original draft, Writing—review & editing.
